# The nephroprotective effects of *Daucus carota* and *Eclipta prostrata* against cisplatin-induced nephrotoxicity in rats

**DOI:** 10.1080/21655979.2021.2009977

**Published:** 2021-12-24

**Authors:** Muhammad Omer Iqbal, Asad Saleem Sial, Imran Akhtar, Muhammad Naeem, Abu Hazafa, Rais A. Ansari, Syed A. A. Rizvi

**Affiliations:** a Shandong Provincial Key Laboratory of Glycoscience and Glycoengineering, School of Medicine and Pharmacy, Ocean University of China, Qingdao, Shandong, China; b Department of Pharmacognosy, Faculty of Pharmacy and Pharmaceutical Sciences, University of Karachi, Karachi, Pakistan; c Department of Pharmacology, Faculty of Pharmacy and Alternative Medicine, The Islamia University of Bahawalpur, Bahawalpur, Pakistan; d College of Life Science, Hebei Normal University, Shijiazhuang, Hebei, China; e Department of Biochemistry, Faculty of Sciences, University of Agriculture, Faisalabad, Pakistan; f Department of Pharmaceutical Sciences, College of Pharmacy, Nova Southeastern University, Fort Lauderdale, FL, USA; g Department of Pharmaceutical Sciences, Hampton University School of Pharmacy, Hampton, VA, USA

**Keywords:** *Daucus carota*, Cisplatin, *Eclipta prostrata*, nephrotoxicity, nephroprotection, rats

## Abstract

The overuse of cisplatin (>50 mg/m^2^) is limited to nephrotoxicity, ototoxicity, gastrotoxicity, myelosuppression, and allergic reactions. The objective of this study was to investigate the nephroprotective effects of *Daucus carota* and *Eclipta prostrata* extracts on cisplatin-induced nephrotoxicity in Wistar albino rats. The study involved male Wistar albino rats of 8 weeks weighing 220–270 g. A single injection of 5 mg/kg was injected into the rats for nephrotoxicity. Rats were divided into four groups based on dose conentrations. Blood and urine samples of rats were collected on the 0, 7^th^, 14^th^, and 21^st^ days for nephrological analysis. The results showed that Cis + DC/Cis + EP (600 mg/kg) significantly (p < 0.001) increased the body weight and reduced the kidney weight of cisplatin-induced nephrotoxicity in rats (p < 0.001) as compared to Cis group. The results showed that 600 mg/kg administration of Cis + DC/Cis +EP successfully (p < 0.005) improved the urine and plasmin creatinine, Na, and K level compared to the Cis group. Histopathological results confirmed that Cis + EP/Cis + DC effectively improved the renal abnormalities. It is concluded that the co-administration of Cis + EP extract showed exceptional nephroprotective effects at a dose rate of 600 mg/kg.

## 1. Introduction

According to emerging evidence, nephrotoxicity is one of the most persistent kidney problems that comprises about 8–15% lifetime risk in Europe, 2–5% in Asia, and 20% in the Middle East. Nephrotoxicity leads to a reduction in glomerular filtration rate and an increase in creatinine and blood urea nitrogen in the serum, ultimately increasing blood pressure and fluid retention in the body (overhydration) [1,2]. The kidney is the primary organ targeted by toxic effects caused by medications. The kidney derives 25% of heat output and is naturally exposed to circulatory drugs and chemicals as central excretion bodies. The overof nephrotoxic drugs also contributes to acute kidney failure and increased morbidity and mortality [3,4]. Because of their functions in glomerular concentrations, drug delivery, and metabolism, the epithelial cells of the renal proximal convoluted tubules (PCT) are a crucial target for nephrotoxicants [5]. Nephrotoxic agents usually damage the renal tubular epithelial cells either by reacting indirectly (through metabolites) or directly with membrane components and cellular macromolecules [6]. Polyene antibiotics (like amphotericin), heavy metals (like Pb and Hg), and organic cations including cationic amino acids and spermine caused direct renal damage by reacting with cholesterol, sulfhydryl groups, and membrane phospholipids, respectively, whereas cysteine conjugates, cisplatin, and acetaminophen formed metabolites such as oxalates and fluoride in hepatic metabolism [1,7].

Cisplatin is the most commonly used and potent chemotherapeutic agent against different solid organ cancers, including head, neck, lung, breast, bladder, and ovary. Besides its multiple advantages, cisplatin also induces several side effects, including ototoxicity, gastrotoxicity, myelosuppression, and allergic reactions [8,9]. According to emerging evidence, the main detrimental effect of cisplatin is the dose-limiting nephrotoxicity that is responsible for mortality and morbidity [10,11]. Several previously published studies linked nephrotoxicity to single doses of cisplatin (50–100 mg/m^2^) [9,12]. Nephrotoxicity caused by cisplatin occurs mainly in the renal proximal convoluted tubules (PCT) [13].

Nephrotoxicity is the most common side effect due to cisplatin accumulation in the kidneys and chemotherapy for treating cancer [14]. Cis-diamminedichloroplatinum(II) (CDDP) therapy leads to the inflammatory pathway reduction of antioxidant levels due to a failure to protect antioxidants from the harm caused by antineoplastic drugs to free radicals. CDDP disturbs the equilibrium between antioxidants and peroxides, and its fibrosis is closely related to a rise in oxidative damage [15,16]. The cisplatin complex moves through the cell membranes in a unionized form due to its high chloride concentration in the plasma. Cl-plasma is exaggerated by the intracellular concentration, and chloride ligands are displaced by water, resulting in a nephrotoxic formation of the positive platinum complexes. The cisplatin molecule binds to the guanine DNA base and inhibits DNA, RNA, and protein synthesis. When cisplatin binds to DNA, it forms an interface and intrastrand, resulting in a faulty genetic code model and halt in its formation and duplication [17,18].

During the past few decades, natural compounds have been considered as one of the promising therapeutic agents against cancer, cardiovascular diseases, aging, diabetes, and especially neurodegenerative disorders due to their high mode of action, efficiency, accuracy, and fewer side effects [19,20]. *Daucus carota L*. (DC) is a root vegetable generally referred to as carrot and commonly cultivated throughout Pakistan. Phenols, polyacetylene, carotenoids, ascorbic acid, and tocopherol are the plenteous phytonutrients found in carrots [21]. It possesses multiple pharmacological properties, including hypotension [22], anti-fertility [23], hepatoprotection [24], anti-spasmodic [25], anti-bacterial, inhibition of monoamine oxidase and cyclooxygenase. Moreover, it has historically been used in treating nephrosis as a nephroprotective agent [26]. *Eclipta prostrata* L. (family, Asteraceae) is commonly known as false daisy or ‘Bhangra Siah’. Phytosterols, β-amyrin, triterpenes such as ecalbatin, equinoxic acid, flavones such as luteolin, and coumarin have been identified in this plant [27]. It showed various pharmacological activities, including hepatoprotective, antimicrobial, antioxidant, anti-inflammatory, analgesic, and antiviral [28].

Although some plants have shown good results against nephrotoxicity-induced cisplatin to date, no approved drug is available on the market to regenerate the renal tubular cells after cisplatin damage. However, based on the remarkable results of natural products regarding nephrotoxicity induced by cisplatin, herbal medicines are gaining the special attention of researchers. The present study investigated the nephroprotective effect of *Eclipta prostrata* (EP) and *Daucus carota* (DC) against cisplatin-induced nephrotoxicity in Wistar albino rats.

## 2. Material and Methods

### 2.1. Chemicals

The analytic rank chemicals including cisplatin (Mylan S.A.S France), 2, 2-diphenyl, 1-picrylhydrazle, formalin, ketamine, and xylazine were purchased from Prix Lab Lahore, Pakistan. Ethanol (99.2% pure), picric acid (99.5% pure), NaOH, and trichloroacetic acid (TCA; 97% pure) were got from Sigma-Aldrich, USA. *Daucus carota* seed and *Eclipta prostrata* leaves were purchased from a local market, Pakistan.

### 2.2. Preparation of plant extract

*Daucus carota* seeds and *Eclipta prostrata* leaves were collected from the local market in Multan, Pakistan. Plants were authenticated by expert taxonomists’ cooperation at the Department of Botany, Bahauddin Zakariya University, Multan, Pakistan, with the voucher number (R.R. Stewart F.W. Pak.708/11) for further reference. *D. carota* seeds and *E. prostrata* leaves were washed and powdered under a blender’s shade for 15 days. 800 g of *D. carota* seeds powder was soaked in a hydroalcoholic solvent (70:30 v/v) in 3 L colorful air-tight amber pots for nine days. After filtration, it was evaporated by a rotary evaporator (Heidolph Laborota 4000 efficient, Germany) at reduced pressure and 30–40 °C. The obtained semi-solid residue was refrigerated before further analysis. The extracts of *D. carota* seed and *E. prostrata* leaves were kept in the refrigerator (−4 °C) for further analysis [29,30].

### 2.3. Animals

Albino Wistar male rats of 6–7 weeks weighing 220–270 g were purchased from a local market and kept in polycarbonate cages that were covered by raw dust and changed every 3 days under normal laboratory conditions (27 ± 2 °C) in the Pharmacology Research Laboratory, Muhammad Institute of Medical and Allied Health Sciences, Multan, Pakistan. The rats were fed with water and standard diet pallets. Male albino Wistar rats were placed in light and darkness for 12 h each. According to the National Research Council, the experiments were performed and approved by the Ethical Committee of the Muhammad Institute of Medical and Allied Science, Multan, Pakistan [31].

### 2.4. Experimental model

A single bolus dose of cisplatin (5 mg/kg) was injected into the rat to induce nephrotoxicity [32]. Rats were typically divided into four different groups of six animals each. Group I administrated with normal saline by the oral route for 21^st^ days and named as control. Group II (nephrotoxicity) received cisplatin alone via intraperitoneal injection of 5 mg/kg on the first day, followed by regular oral saline until the 21^st^ day. Group III was given the cisplatin + extract of each plant (DC and EP) in the amount of 400 mg/kg, while Group IV received the dose of cisplatin + extract of each plant (DC and EP) in the amount of 600 mg/kg. Daily food and water consumption were regularly measured along with the body weights and glucose levels of rats. The body and kidney weight of rats were measured at the beginning and end of the experiment.

### 2.5. Measurement of plasma/urine Na and K level

Sodium (Na) and potassium (K) concentrations in plasma and urine were measured by using a flame photometer (Sherwood Model 410, UK). The samples were diluted at 1:200 for the measurement of Na in urine and plasma samples and K in plasma samples, while 1:1000 was made to measure K in urine samples. All the samples were measured in triplicate. The urine was collected after 0, 7^th^, 14^th^, and 21^st^ days from all experimental groups of rats by placing each rat on a plastic dish for sodium (Na) and potassium (K) level analysis [33]. The rats were kept for 24 h in metabolic cages with free access to tap water and measured the total intake of water and urine exit. The collected urine samples were reserved at −30 °C for the estimation of Na, and K levels. Similarly, the blood samples of all experimental groups were collected at 0, 7^th^, 14^th^, and 21^st^ days in EDTA tubes and centrifuged at 2300 × g to remove the plasma for the estimation of Na and K levels. The plasma was extracted and stored at −80 °C for further analysis.

The urine flow rate is the amount of urine excreted per unit time and it was calculated using the following Eq. (1) in µL/min/100 g of BW.

(1) $$Urine\;flow\;rate\;\left(\mu L/min/100g\;of\;BW\right)=\;\frac{Urine\;\;output\;\left(\mu \frac{L}{24}h\right)\times 1000}{1400\times rat\;\;weight\;\left(g\right)}\times 100$$

### 2.6. Measurement of plasma and urine creatinine level

Plasma and urinary creatinine concentrations were measured spectrophotometrically (Jaffe’s reaction) by the method of Seeling and Wust [34] with slight modifications. Urinary samples were diluted up to 50 times with distilled water. Both plasma and urine samples were deproteinized using Trichloroacetic acid (1.2 M/L) along with centrifugation, and the supernatant was used for the measurement. The principle of this assay is based on the reaction between creatinine in the sample and picric acid in an alkaline medium to form a colored complex. This complex can be detected by a spectrophotometer at a 520 nm wavelength. To avoid the interferenece, the complex formation was measured in a short period of time after preparation. The total volume of the sample, blank and standard was transferred to a 96-well microtiter plate and incubated for 20 min at room temperature. Following the incubation period, the absorbance of the mixture was measured using a microplate reader (Synergy HT BioTek ® USA). All the samples were analyzed in triplicate and the concentration of creatinine in plasma and urine was calculated using the following Eqs. (2,3):

(2) $$Plasma\;creatinine\left(\frac{mg}{dl}\right)=\;\frac{Abs.of\;sample-Abs.of\;blank}{Abs.of\;standard-Abs.of\;blank}Conc.of\;standard$$

(3) $$Urine\;creatinine\left(\frac{mg}{dl}\right)=\;\frac{Abs.of\;sample-Abs.of\;blank}{Abs.of\;standard-Abs.of\;blank}Conc.of\;standard\times 50$$

### 2.7. Histopathological analysis

The histopathological analysis of the kidney was performed using Olympus light microscopy (X400) with an Olympus digital camera. First of all, the formalin kidneys were dehydrated, cleared in xylene, and placed in paraffin. Next, the kidneys were cut into 5 µm sections and stained with periodic acid schief and hematoxylin. Specific sections (5 µm each) of the kidneys were rehydrated and desalinized with alcohol. Next, these sections were treated with a particular amount of endogenous peroxidases for 0.5 h at 37 °C and rinsed three times in phosphate buffer saline (PBS) at pH 7.00. After rinsing, the kidney sections were heated with 0.01 M sodium citrate buffer at pH 6.00 for 25 min and incubated for 1 h with 1% BSA. After incubation, the sections were condensed with hematoxylin, dehydrated with alcohol, and cleaned with xylene [35,36]. After incubation, the sections were condensed with hematoxylin, dehydrated with alcohol, and cleaned with xylene [35,36].

### 2.8. Statistical analysis

One-way analysis of variance (ANOVA) and Bonferroni’s all-mean as a post hoc analysis were performed for all the data with a Mean ± SEM (n = 6) and methodological significance between different experimental groups. Statistically, significance was considered as p < 0.05 [33].

## 3. Results

The study aimed to determine the alterations in biochemical and histopathological parameters in cisplatin-induced nephrotoxicity and its protection by treatment with hydro-alcoholic extracts of *Daucus carota* and *Eclipta prostrata*. Acute nephrotoxicity was induced by cisplatin (5 mg/kg) in Wistar rats. Nephrotoxic rats were treated with *Daucus carota* and *Eclipta prostrata* hydroalcoholic extracts (400 and 600 mg/kg body weight) by oral gavage. Cisplatin treatment elevated (P < 0.05) the levels of urine flow rate, urine output, urinary sodium, and potassium. but lowered (P < 0.05) body weight, plasma sodium and potassium as compared to the control. Pre and post-treatment with plant extract at 400 and 600 mg/kg attenuated the altered levels of various enzymatic and oxidative parameters in blood and renal tissue in a dose-dependent manner. The extract attenuated the degenerative and necrotic changes of proximal convoluted tubules induced by cisplatin which initiated the good nephroprotective potential of *Daucus carota* and *Eclipta prostrata* extracts. The details are presented in the following sections.

### 3.1. Effect of DC and EP extracts on body and kidney weight of Cis treated rats

Nephrotoxicity is initiated by a solitary dosage of cisplatin (5 mg/kg i.p) that is biochemically shown by escalation (P ≤ 0.05) in body weight, urine output, urinary sodium, urinary potassium, plasma creatinine, and kidney weight. In contrast, cisplatin causes a decrease (P ≤ 0.05) in plasma Na and K and urinary creatinine. Table 1 shows the results of body and kidney weight between and after the administration of cisplatin and DC/EP extracts. The activities were measured on the 0, 7^th^, 14^th^, and 21^st^ days after dose administration. Results reported that the bodyweight of rats was 246 ± 7.9, 183 ± 8.9, 197 ± 9, and 162 ± 8.3 g at 0, 7^th^, 14^th^, and 21^st^ days, respectively, after 5 mg/kg cisplatin (cis) administration. It was noted that after 400 mg/kg administration of cis + DC extract, the body weight was 219 ± 7.9, 245 ± 8.6, and 228 ± 10 g, while with cis + EP extract, the body weight was 222 ± 8.1, 243 ± 8.5, and 231 ± 13 g after 7^th^, 14^th^, and 21^st^ days, respectively. The body weight was observed to increase up to the 14th day, after which it began to decrease, and the maximum BW was observed after the 14th days of observation with both plant extracts. It was also noted that higher dose of administration of extract increased the BW of a rat compared to lower dose concentration.

**Table 1.** Effect of crude extracts of DC and EP on body weight of cisplatin-treated rats

Body weight (g)	Observation (day)			
Groups	0	7^th^	14^th^	21^st^
*Daucus carota* (DC)				
Control	242 ± 7.5	264 ± 8.2	326 ± 9.0	314 ± 8.5
Cis	246 ± 7.9*	183 ± 8.9**	197 ± 9**	162 ± 8.3**
Cis + DC^a^	265 ± 8.4*	219 ± 7.9**	245 ± 8.6**	228 ± 10**
Cis + DC^b^	267 ± 8.6*	228 ± 5.5**	282 ± 8.4**	268 ± 9.1**
*Eclipta prostrata* (EP)				
Control	242 ± 7.5	264 ± 8.2	326 ± 9.0	314 ± 8.5
Cis	246 ± 7.9*	183 ± 8.9**	197 ± 9**	162 ± 8.3**
Cis + EP^a^	261 ± 8.2*	222 ± 8.1**	243 ± 8.5**	231 ± 6.2**
Cis + EP^b^	265 ± 8.4*	231 ± 5.8**	284 ± 8.7**	272 ± 9.5**

Mean ± SD (n = 6), where Cis is cisplatin (5 mg/kg i.p), Cis + DC^a^ is cisplatin + *Daucus carota* extract (400 mg/kg/21 days), Cis + DC^b^ is cisplatin + *Daucus carota* extract (600 mg/kg/21 days. Cis + EP^a^ is cisplatin + *Eclipta prostrata* extract (400 mg/kg/21 days) and Cis + EP^b^ is cisplatin + *Eclipta prostrata* extract (600 mg/kg/21 days). *p < 0.005 and **p < 0.05.

**Figure 1.**
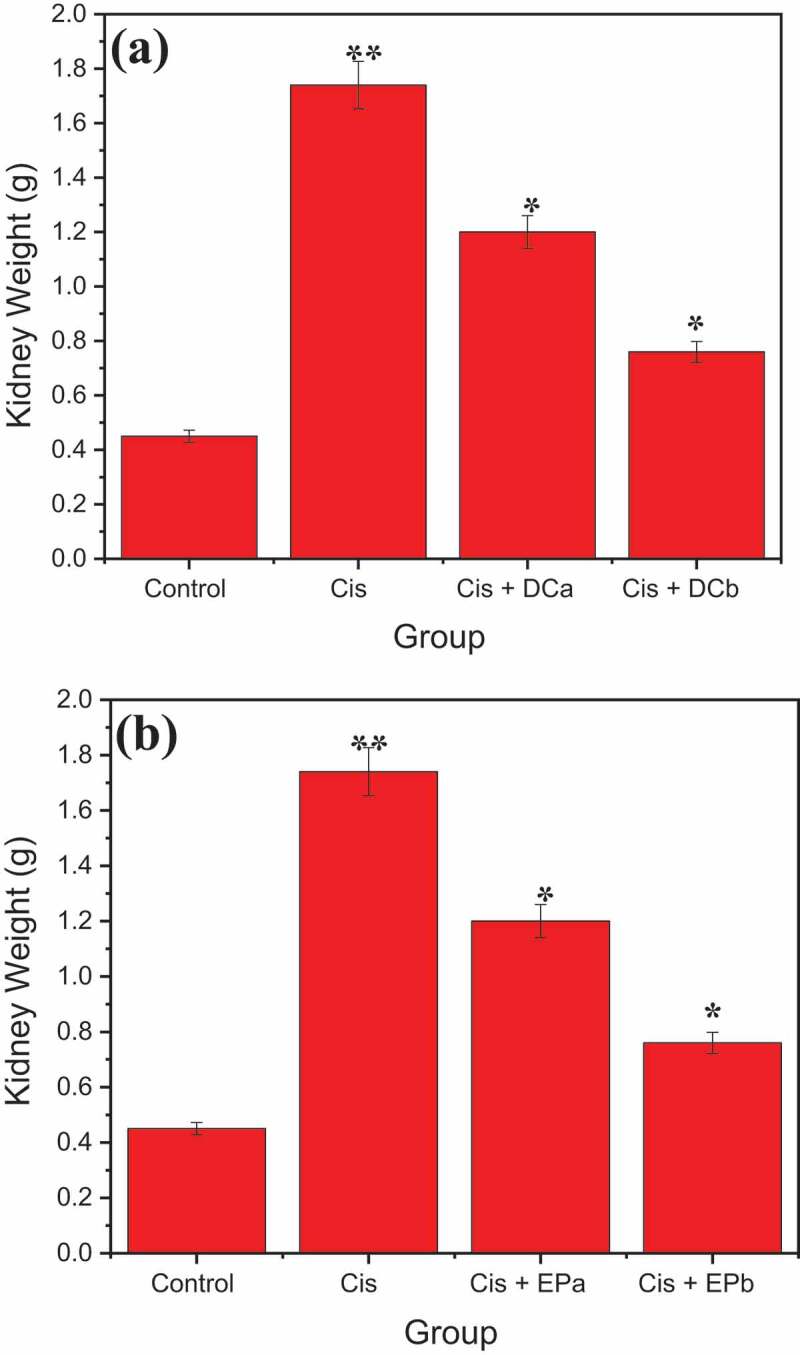
Effect of (a) DC and (b) EP extracts on kidney weight in cisplatin-treated rats. Where Cis is cisplatin (5 mg/kg i.p), Cis + DC^a^ is cisplatin + *Daucus carota* extract (400 mg/kg/21 days), Cis + DC^b^ is cisplatin + *Daucus carota* extract (600 mg/kg/21 days. Cis + EP^a^ is cisplatin + *Eclipta prostrata* extract (400 mg/kg/21 days) and Cis + EP^b^ is cisplatin + *Eclipta prostrata* extract (600 mg/kg/21 days). *p < 0.001 and **p < 0.05

Figure 1 shows the results of kidney weight loss after the administration of cisplatin and DC and EP plant extracts. Results reported that the administration of cisplatin increased the kidney weight by about 1.74 ± 0.090 g that was reduced to 1.2 ± 0.045 g after co-administration of cisplatin + DC extract at 400 mg/kg, and 0.76 ± 0.043 g after co-administration of cisplatin + DC extract at 600 mg/kg. Similarly, 1.3 ± 0.0046 and 0.74 ± 0.044 g kidney weight loss was observed after co-administration of cisplatin + EP extract at 400 and600 mg/kg/7 days, respectively. Overall, it was concluded that 600 mg/kg administration of cisplatin with both plant extracts significantly reduced the kidney weight loss compared to 400 mg/kg administration.

### 3.2. Effect of DC and EP extracts on urine output of Cis treated rats

**Figure 2.**
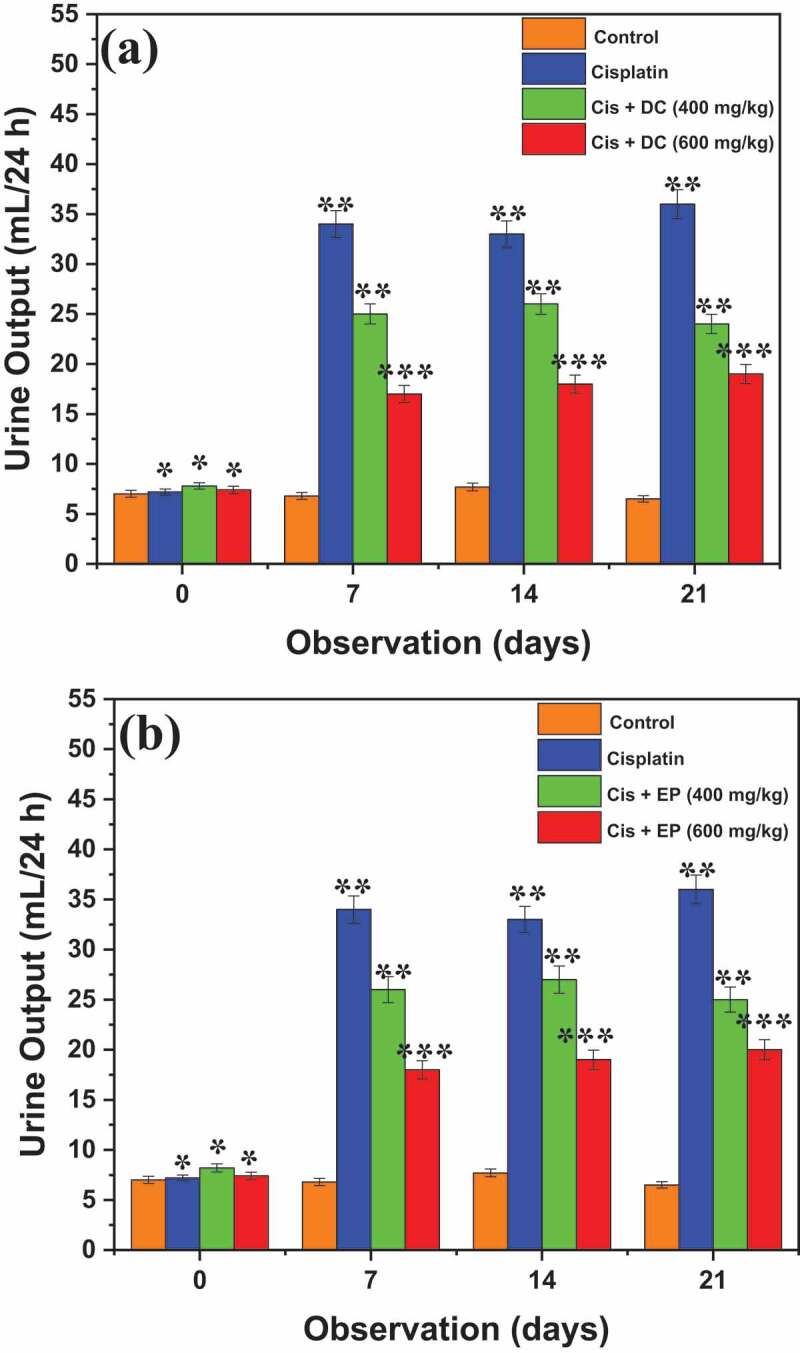
Representation of effect of (a) DC and (b) EP extracts on body urine output in cisplatin-treated rats. The results showed that co-administration of Cis + DC/Cis + EP significantly reduced the body urine output at a dose administration of 600 mg/kg. *p < 0.001, **p < 0.05, and ***p < 0.005

Figure 2(a,b) represents the effect of DC and EP extracts on the urine output of cisplatin-treated rats. Results were observed after 0, 7^th^, 14^th^, and 21^st^ days of dose administration. It was noted that control showed urine output of 7.0 ± 1.7, 6.8 ± 1.7, 7.7 ± 1.7, and 6.5 ± 1.2 mL/24 h after 0, 7^th^, 14^th^, and 21^st^ day of observations, respectively. Similarly, cisplatin-treated rats represented the urine output of 7.2 ± 2.0, 34 ± 2.4, 33 ± 3.2, and 36 ± 1.7 mL/24 h after 0, 7^th^, 14^th^, and 21^st^ day of observations, respectively. The co-administration of Cis + DC at 400 mg/kg revealed the urine output of 7.8 ± 1.6, 25 ± 1.9, 26 ± 8.4, and 24 ± 1.3 mL/24 h, while Cis + EP at 400 mg/kg showed the urine output of 7.8 ± 1.6, 25 ± 1.9, 26 ± 8.4, and 24 ± 1.3 mL/24 h after, 7^th^, 14^th^, and 21^st^ day of observations, respectively. In addition, at 600 mg/kg, co-administration of Cis + EP presented the maximum urine output of 20 ± 0.90 mL/24 h after 21^st^ days of observation. In contrast, Cis + DC showed the maximum urine output of 20 ± 0.90 mL/24 h at the same parameter. Overall, cisplatin-treated rats produced more urine; however, co-administration of Cis+ DC/Cis+ EP significantly reduced urine output from 36 1.7 to 18 1.6 mL/24 h at a dose rate of 600 mg/kg. However, it was determined that Cis+ DC/Cis+ EP at a dose rate of 600 mg/kg showed better results than 400 mg/kg.

### 3.3. Effect of DC and EP on urinary Na and K in Cis treated rats

**Figure 3.**
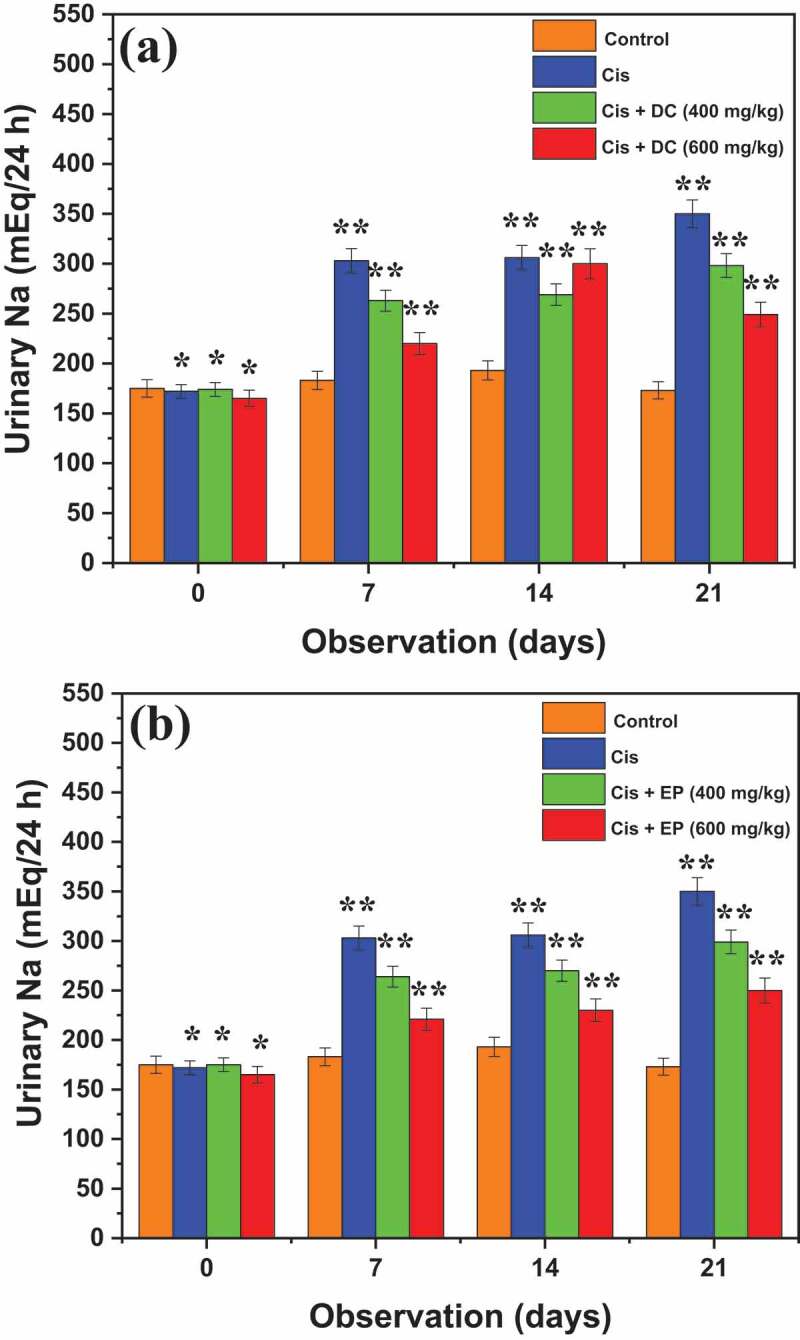
Histogram of the effect of (a) DC and (b) EP extracts on urinary sodium in cisplatin-treated rats. The results showed that co-administration of Cis + DC/Cis + EP significantly reduced the body urinary Na at the dose administration of 600 mg/kg. *p < 0.001, **p < 0.05

Figure 3(a,b)shows the results of the effect of DC and EP plant extracts on urinary sodium (Na) in cis-treated rats. The readings were noted after 0, 7^th^, 14^th^, and 21^st^ days of dose administration. Results reported that after 7^th^ day of observation, cis-treated rats, Cis + EP (400 mg/kg) and Cis + EP (600 mg/kg) showed the urinary Na concentration of 303 ± 13, 264 ± 8.3, and 221 ± 8.5 mEq/24 h respectively, while Cis + DC (400 mg/kg) and Cis + DC (600 mg/kg) revealed the urinary Na concentration of 263 ± 8.1, 220 ± 8.4 mEq/24 h, respectively. Similarly, after 21^st^ days of observation, Cis-treated rats, Cis + EP (400 mg/kg) and Cis + EP (600 mg/kg) displayed the urinary Na concentration of 350 ± 19, 299 ± 13, and 250 ± 8.0 mEq/24 h, respectively, whereas Cis + DC (400 mg/kg) and Cis + DC (600 mg/kg) revealed the urinary Na concentration of 298 ± 12 and 249 ± 7.9 mEq/24 h, respectively.

**Figure 4.**
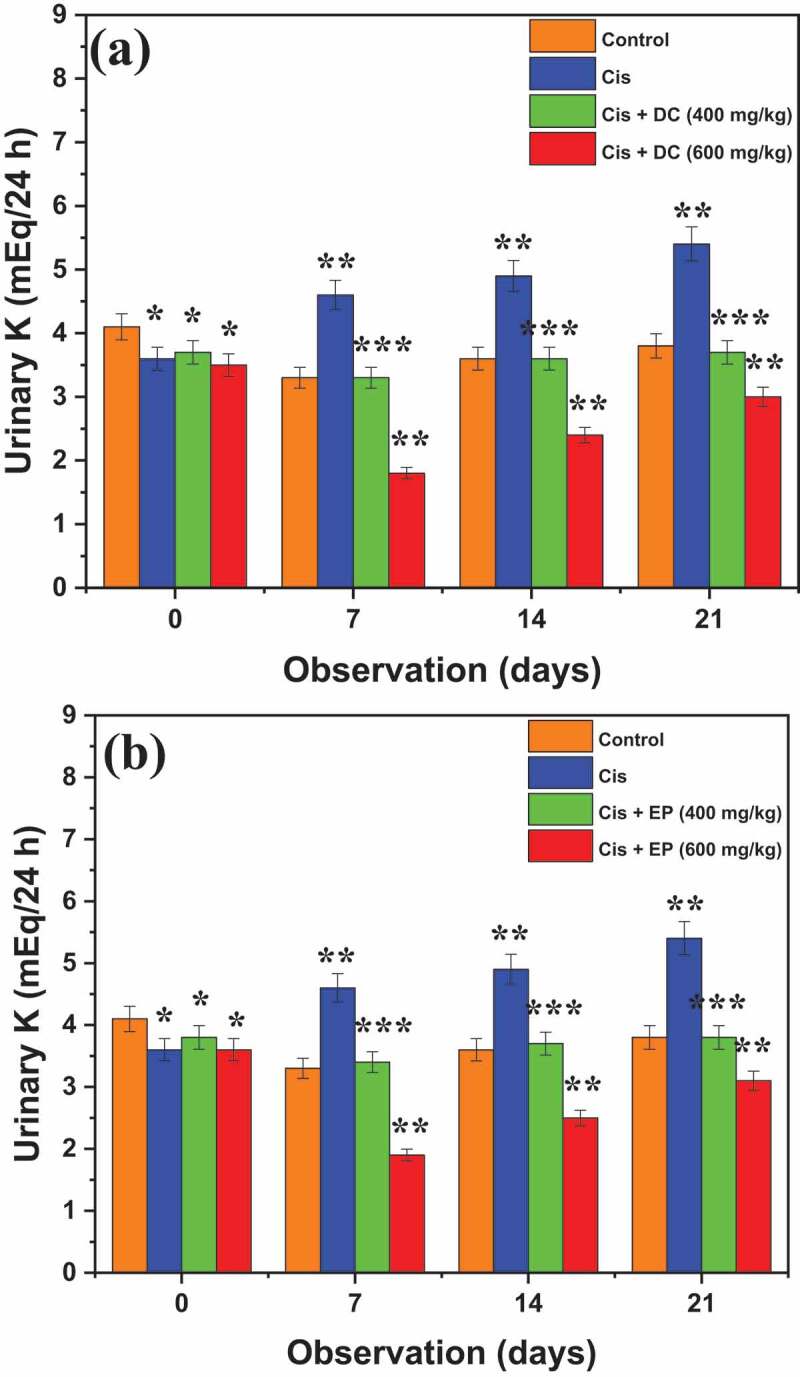
Effect of (a) DC and (b) EP plant extracts on urinary potassium in cisplatin-induced nephrotoxic rats. *p < 0.001, **p < 0.05, and ***p < 0.005

Figure 4(a,b) demonstrates the results of the effect of DC and EP plant extracts on urinary potassium (K) in cis-treated rats. It was noted that Cis-treated rats showed urinary K concentrations of 3.5 ± 0.37, 4.5 ± 0.20, 4.9 ± 0.30, and 5.3 ± 0.35 mEq/24 h after 0, 7^th^, 14^th^, and 21^st^ days of observation. Results reported that co-administration of Cis + DC at the rate of 400 mg/kg significantly reduced the concentration of K from 5.3 ± 0.35 to 3.3 ± 0.21 mEq/24 h after 7^th^ days of observation, while at a dose administration of 600 mg/kg, Cis + DC showed more impressive results (1.8 ± 0.21 mEq/24 h) regarding the reduction of K from the urine of nephrotoxic rats. A similar trend was observed in the co-administration of Cis + EP. Overall, it is concluded that Cis + EP/Cis + DC co-administration at the rate of 600 mg/kg significantly reduced the potassium level in the urine of cis-treated rats.

### 3.4. Effect of DC and EP on plasma Na and K in Cis treated rats

**Figure 5.**
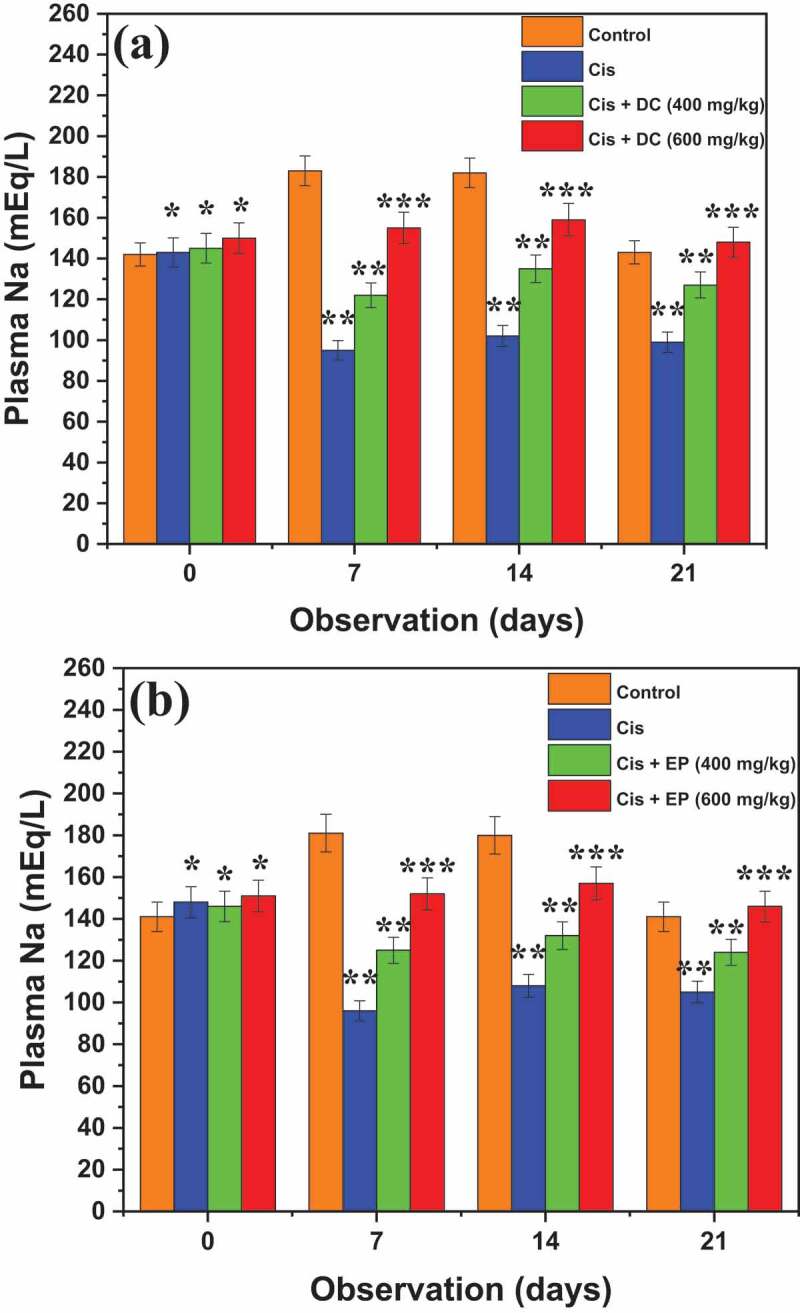
Representation of effect of (a) DC and (b) EP plant extracts on plasmin sodium in cisplatin-induced nephrotoxic rats. *p < 0.001, **p < 0.05, and ***p < 0.005

To further analyze the effect of DC and EP extracts, the levels of plasmin potassium (K) and sodium (Na) were determined in each group of cis-treated rats. Figure 5(a,b) represents the results of the effect of DC and EP extracts on plasmin Na. Results reported that Cis treated group showed a significant reduction in the level of plasmin Na from the first day to 21^st^ day of observations (143 ± 4.7 to 99 ± 6.3 mEq/L) as compared to control that noted as 142 ± 5.2 mEq/L on the first day and 143 ± 6.4 mEq/L on 21^st^ day of observations. Results revealed that the 400 mg/kg co-administration of Cis + DC significantly improved the plasmin Na level of 122 ± 5.7 mEq/L after the 7^th^ day of observation that increased to 127 ± 7.5 mEq/L after 21^st^ days of observation as compared to the Cis group. On the other hand, 400 mg/kg co-administration of Cis + EP extract effectively improved the plasmin Na level of 125 ± 7.5 mEq/L after the 7^th^ day of observation, which enhanced to 128 ± 7.3 mEq/L after 21^st^ day of observations.

Similarly, administration of Cis + DC and Cis + EP extracts (600 mg/kg) showed a maximum improvement of 159 ± 6.2 and 157 ± 5.9 mEq/L, respectively as compared to the Cis group. Results indicated that 600 mg/kg co-administration of Cis with both plant extracts showed a remarkable elevated level of plasmin Na as compared to Cis group. However, the treatment of Cis-effect rats showed a significant result with a 600 mg/kg dose of co-administration of Cis with both plant extracts.

**Figure 6.**
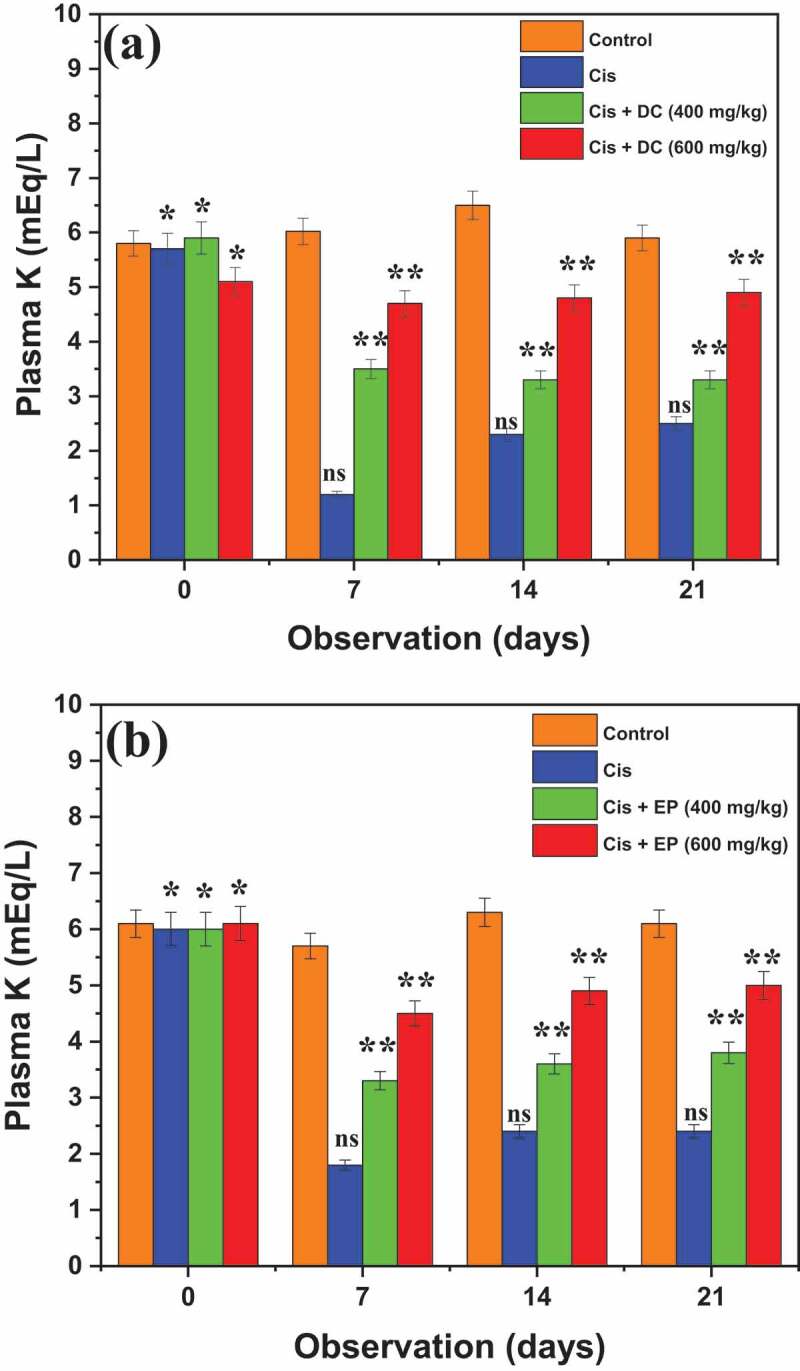
Histogram of the effect of (a) DC and (b) EP extracts on plasmin potassium in cisplatin-treated rats. The results showed that co-administration of Cis + DC/Cis + EP significantly reduced the body urine output at dose administration of 600 mg/kg. *p < 0.005, **p < 0.05, and ns = non-significant

Figure 6(a,b) presents the nephroprotective effects of DC and EP extract on plasmin K levels in each group of rats. Results demonstrated that the Cis group showed the K level of 5.7 ± 0.18 mEq/L after the first day that decreased to 2.5 ± 0.29 mEq/L due to nephrotic effects. It is observed that 400 mg/kg administration of Cis + DC and Cis + EP showed a significant elevation in plasmin K level of about 3.5 ± 0.22 and 3.3 ± 0.23 mEq/L after 7^th^ days, which increased to 3.4 ± 0.23 and 3.8 ± 0.19 mEq/L after 21^st^ days of observation as compared to Cis group that indicated that Cis with plant extracts showed the better nephroprotective results. Similarly, co-administration of Cis + DC/Cis + EP at 600 mg/kg concentration presented more remarkable results regarding the elevation of plasmin K level as compared to the Cis group. Overall, it is concluded that Cis + DC/Cis + EP at 600 mg/kg concentration showed better results as compared to the Cis group and 400 mg/kg concentration.

### 3.5. Effect of DC and EP on urinary and plasma creatinine in Cis treated rats

**Figure 7.**
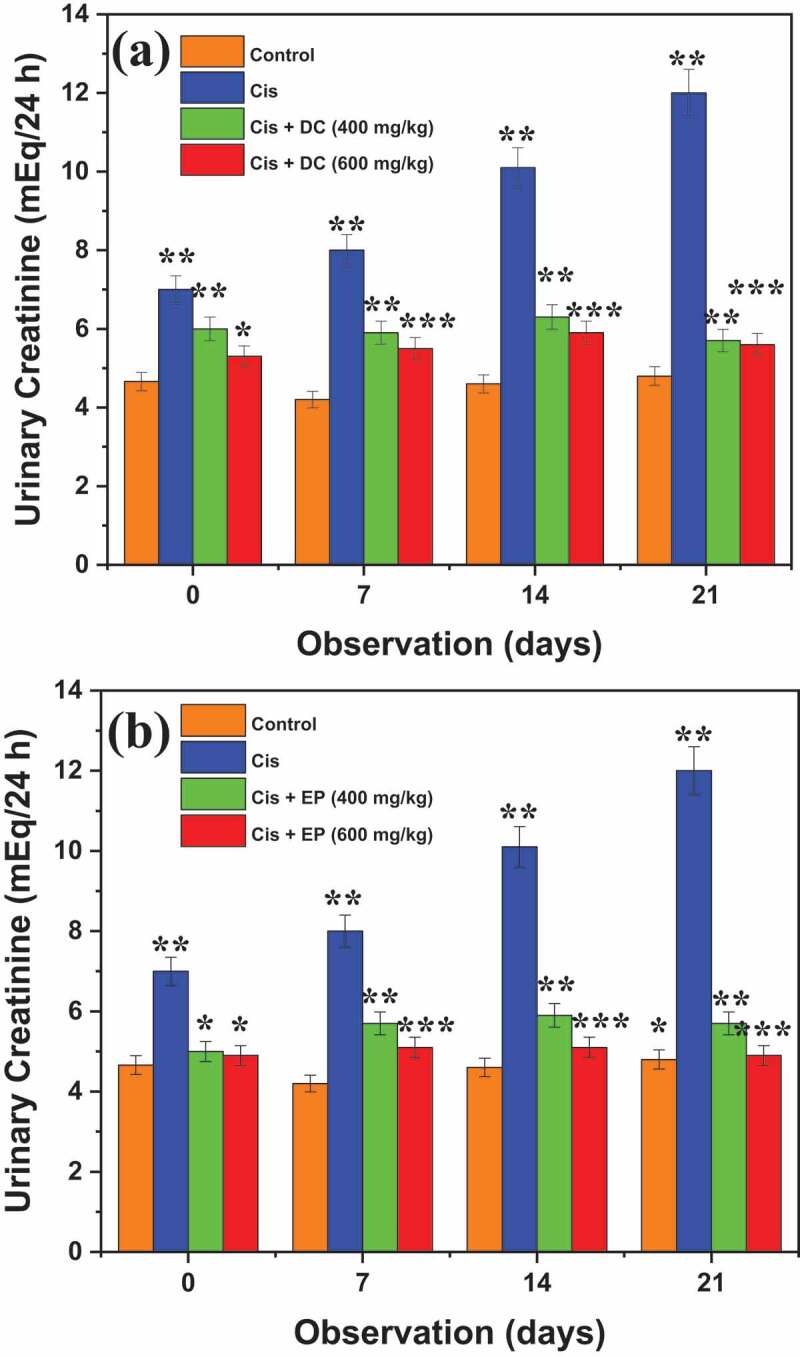
The representation of (a) DC and (b) EP plant extracts on urinary creatinine in cisplatin-induced nephrotoxic rats. *p < 0.001, ** p < 0.05, and ***p < 0.005

**Figure 8.**
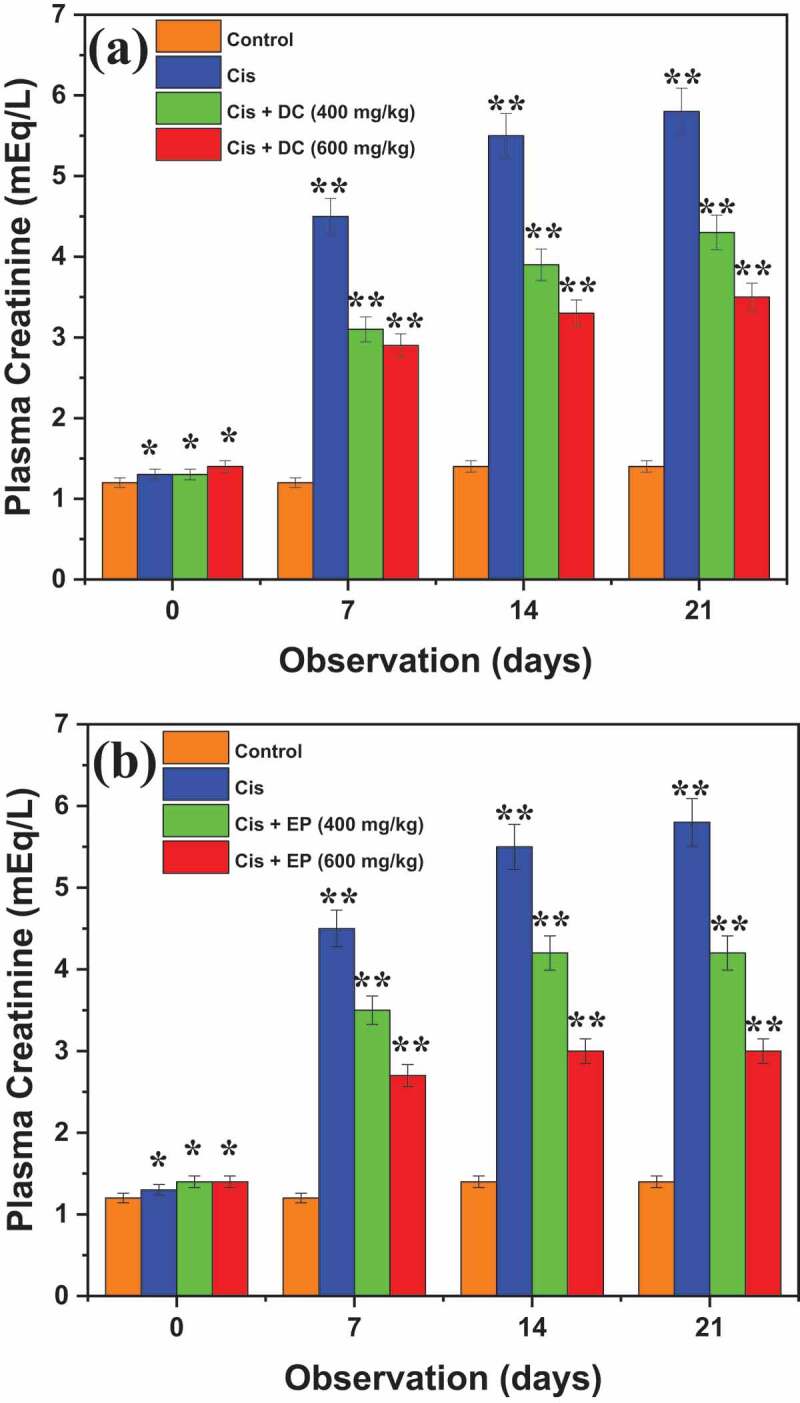
Interpretation of effect of (a) DC and (b) EP plant extracts on plasmin creatinine in cisplatin-induced nephrotoxic rats. *p < 0.001, ** p < 0.05, and ***p < 0.005

Figure 7(a,b) shows the nephroprotective effects of DC and EP on urinary creatinine in cis-treated rats. Results revealed that control (4.8 ± 0.50 mEq/24 h) and cis-treated rats (12 ± 0.35 mEq/24 h) showed an elevated urinary creatinine level. It is observed that Cis + DC/Cis +EP co-administration at the rate of 600 mg/kg significantly reduced the urinary creatinine level of about 5.1 ± 0.24 and 5.5 ± 0.25 mEq/24 h after 7^th^ days, 5.1 ± 0.34 and 5.9 ± 0.26 mEq/24 h after 14^th^ days, and 4.9 ± 0.18 and 5.6 ± 0.25 mEq/24 h after 21^st^ days as compared to cisplatin-treated rats. It is indicated that 600 mg/kg dose of Cis + DC/Cis +EP significantly reduced the urinary creatinine level as compared to Cis group and 400 mg/kg concentration. On the other hand, Figure 8(a,b) presented the results of DC and EP on plasmin creatinine in Cis-treated rats. Results revealed that co-administration of Cis + EP extract at the rates of 400 and 600 mg/kg effectively reduced the plasmin creatinine levels of 4.2 ± 0.30 and 3.0 ± 0.23 mEq/L, respectively, while co-administration of Cis + DC extract at the rates of 400 and 600 mg/kg effectively reduced the plasmin creatinine levels of 4.3 ± 0.31 and 3.5 ± 0.25 mEq/L respectively. Results indicated that Cis + both plant extracts showed better creatinine reduction results than Cis group, which suggested that plant extracts have a positive response concerning nephroprotective effects in Cis treated rats.

### 3.6. Impact of DC and EP extracts on urine flow rate in Cis treated rats

Table 2 shows the nephroprotective results of DC and EP plant extracts on urine flow rate in each group. Based on the results, it was noted that control showed the urine flow rate of 2.3 ± 0.56, 2.1 ± 0.45, 1.7 ± 0.45, and 1.9 ± 0.43 µL/min/100 g of BW after 0, 7^th^, 14^th^, and 21^st^ days, respectively, while Cis group exhibited the urine flow rate of 2.2 ± 0.43, 14 ± 1.3, 14 ± 1.2, and 16 ± 1.2 µL/min/100 g of BW after 0, 7^th^, 14^th^, and 21^st^ days, respectively. It was observed that co-administration of Cis + EP extract at the rate of 400 and 600 mg/kg efficiently condensed the urine flow rate of 7.6 ± 0.69 and 5.3 ± 0.75 µL/min/100 g of BW at 7^th^ days, 7.0 ± 0.50 and 4.0 ± 0.44 µL/min/100 g of BW at 14^th^ days, and 6.8 ± 0.56 and 3.9 ± 0.38 µL/min/100 g of BW at 21^st^ days, respectively, while co-administration of Cis + DC extract at the rate of 400 and 600 mg/kg effectively reduced the plasmin creatinine level of 7.4 ± 0.53 and 4.8 ± 0.41 µL/min/100 g of BW at 14^th^ days, and 6.5 ± 0.60 and 4.2 ± 0.37 µL/min/100 g of BW at 21^st^ days, respectively. Overall, it is concluded that Cis +EP extract (600 mg/kg) showed better results regarding the urine flow in Cis treated rats as compared to Cis +DC extract and Cis group.

**Table 2.** The nephroprotective effect of DC and EP extract on urine flow rate in Cis treated rats

Urine flow rate (µL/min/100 g of BW)		Observation (day)			
Groups	0		7^th^	14^th^	21^st^
*Daucus carota* (DC)					
Control	2.3 ± 0.56		2.1 ± 0.45	1.7 ± 0.39	1.9 ± 0.43
Cis	2.2 ± 0.43		14 ± 1.3**	14 ± 1.2***	16 ± 1.2*
Cis + DC^a^	2.5 ± 0.46		7.9 ± 0.63**	7.4 ± 0.53***	6.5 ± 0.60*
Cis + DC^b^	2.0 ± 0.56		6.1 ± 0.78**	4.8 ± 0.41***	4.2 ± 0.37*
*Eclipta prostrata* (EP)					
Control	2.3 ± 0.56		2.1 ± 0.45	1.7 ± 0.39	1.9 ± 0.43
Cis	2.2 ± 0.43		14 ± 1.3**	14 ± 1.2***	16 ± 1.2*
Cis + EP^a^	2.3 ± 0.45		7.6 ± 0.69**	7.0 ± 0.50***	6.8 ± 0.56*
Cis + EP^b^	1.8 ± 0.54		5.3 ± 0.75**	4.0 ± 0.44***	3.9 ± 0.38*

Mean ± SD (n = 6), where Cis is cisplatin (5 mg/kg i.p), Cis + DC^a^ is cisplatin + *Daucus carota* extract (400 mg/kg/21 days), Cis + DC^b^ is cisplatin + *Daucus carota* extract (600 mg/kg/21 days. Cis + EP^a^ is cisplatin + *Eclipta prostrata* extract (400 mg/kg/21 days) and Cis + EP^b^ is cisplatin + *Eclipta prostrata* extract (600 mg/kg/21 days). *p < 0.001, **p < 0.005, and ***p < 0.05.

### 3.7. Histopathological effects of DC and EP extracts in Cis treated rats

**Figure 9.**
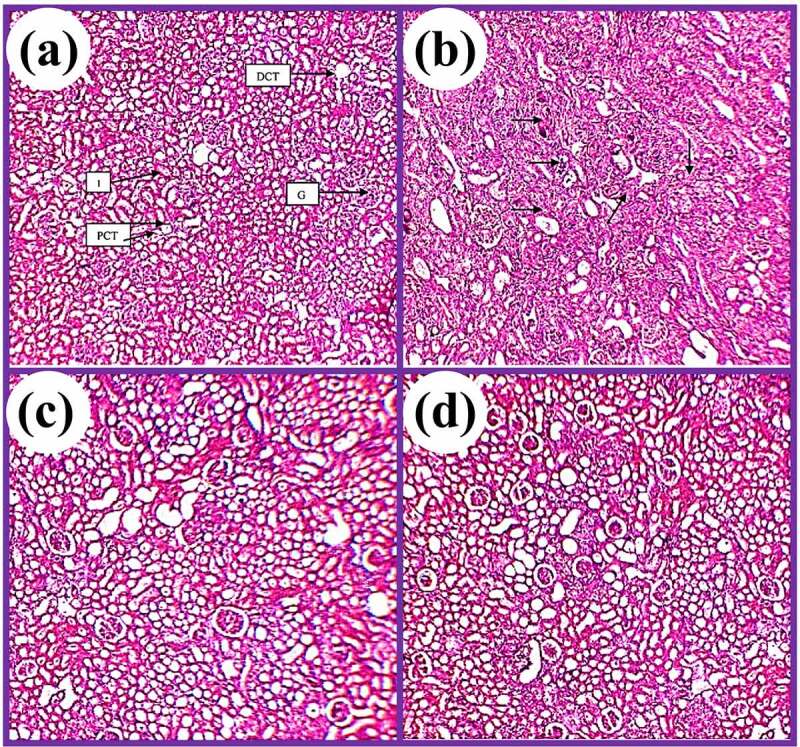
Photomicrograph of histopathological analysis of (a) control, (b) Cis group, (c) Cis + EP (400 mg/kg), and (d) Cis + EP (600 mg/kg)

**Figure 10.**
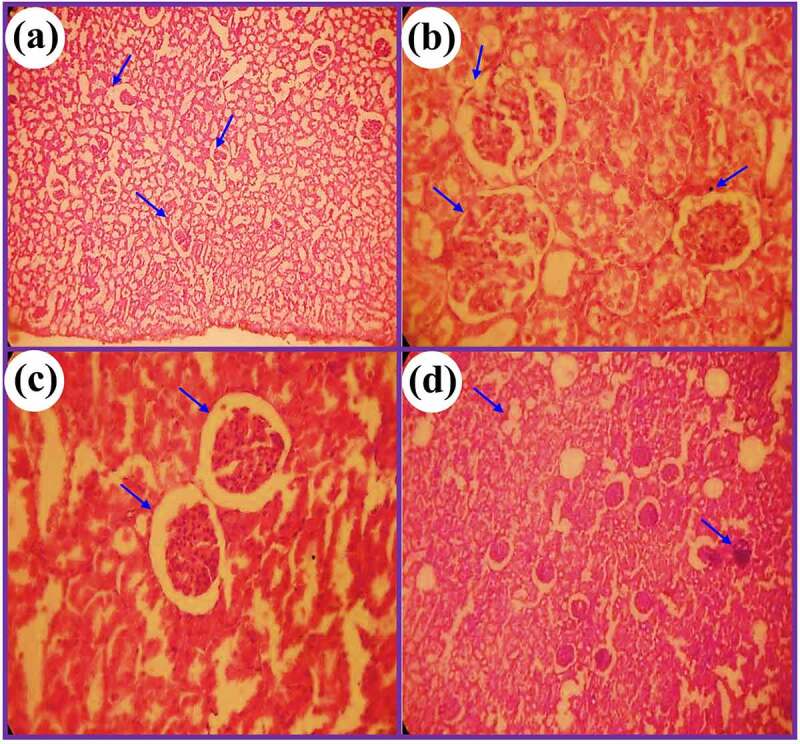
Photomicrograph of kidney tissues of rats for (a) control, (b) Cis group, (c) Cis + DC (400 mg/kg), and (d) Cis + DC (600 mg/kg)

Figures 9 and Figure 10 show the histopathological results of EP and DC extracts in cis-treated rats. The histopathological effects of both plant extracts were examined for H&E stain of histopathological abnormalities using a light microscope. Results showed that the kidney slides of control displayed uniform tubules and normal glomeruli covered with an epithelial layer and showed no capillary obstruction, bleeding, or interstitial damage. While in Cis group, severe tube and glomerular degeneration alongside putrefaction were observed. Besides, several degenerative changes were also observed in Cis group in the form of atrophy lining with tubular and eosinophilic casts in cytoplasmic vacuolization of cells. In addition, glomerular hypertrophy was also noted in cis-treated rats. However, Cis + EP/Cis + DC co-administration at a rate of 400 mg/kg showed normal glomeruli with mild histopathological results of damage in tubules. On the other hand, Cis + EP/Cis + DC co-administration at the rate of 600 mg/kg significantly reduced the histopathological abnormalities induced by Cis, and more minor renal damage was found in the proximal and distal tubule as compared to Cis + EP^a^/Cis + DC^a^.

## 4. Discussion

The main objective of the present study was to investigate the nephroprotective effects of *Daucus carota* (DC) seeds extract and *Eclipta prostrata* (EP) leaves extract against cisplatin-induced nephrotoxic albino Wistar rats. For the first time, a comprehensive study was conducted that explained the nephroprotective effects of DC and EP extracts. The albino rats were divided into four different groups: Group I contained only saline and was designated as control, Group II received cisplatin (5 mg/kg i.p), Group III received co-administration of Cis + DC/Cis + EP (400 mg/kg), and Group IV received co-administration of Cis + DC/Cis + EP (600 mg/kg). The urinary samples were taken within 24 h of administration of dose after 0, 7^th^, 14^th^, and 21^st^ days for urinary creatinine, sodium (Na), potassium (K), and urine flow rate analysis. While blood samples were taken after 0, 7^th^, 14^th^, and 21^st^ days of dose administration for the plasmin analysis of plasmin creatinine, sodium (Na), and potassium (K) analysis. The body weight and kidney index were also investigated after dose administration in each rat group. On the other hand, histopathological analysis of the kidney was also performed to confirm the effect of DC and EP extracts on the nephrotoxicity of rats.

Cisplatin is one of the most commonly used platinum-containing antineoplastic drugs that is used in the treatment of solid tumors including breast, lung, head, and neck [9,37]. Despite its numerous advantages in cancer treatment, its applications are limited to nephrotoxicity, neuro-, and oto- gastrointestinal damage [38,39]. With increasing drug use, drug-based nephrotoxicity has been growing day by day, that causing almost 26% of acute kidney injuries (AKI) [40]. The emerging evidence suggests that a single dose of cisplatin up to 50 mg/m^2^ induces side effects in the kidney. However, according to an estimation across 40% of patients, who received cisplatin doses over this limit and suffered from acute or mild renal dysfunction [39,41]. It was observed that cisplatin-induced nephrotoxicity leads to renal vasculature that alters renal hemodynamics [42].

Cisplatin (Cis) accumulates preferentially in the S3 portion of the proximal renal tubules. It is modified by intracellular hydration to form a reactive metabolite and alters the expression of many water channels and membrane transporters to inhibit the function of mitochondria, which ultimately blocks ATP production and leads to nitrosative and oxidative stress [43]. These pharmacological effects lead to the reabsorption and uncoupling of water that precedes the excretion of electrolytes, including magnesium (Mg), sodium (Na), calcium (Ca), and potassium (K). Moreover, cisplatin attracts different organelles and interfaces in DNA replication that proceed to alter several biological mechanisms, including necrosis and apoptosis, inflammation, and tubular derangement [9,44]. The mechanisms by which Cis causes nephrotoxicity are complex and intervened by different biological pathways involve oxidative stress, apoptosis, and inflammation [45]. ROS production is increased by cisplatin by mitochondria; NADPH oxidase and cellular xanthine oxidase systems are involved in the pathogenesis of Cis induced severe kidney failure [17]. The function of various renal antioxidant enzymes, including CAT, GHx, and SOD are also reduced by cisplatin [46]. However, based on the side effects of cisplatin, there is a need to develop a drug to reduce the pathophysiology of cisplatin. Nowadays, a mixture of different chemicals and natural products are used as potential cisplatin neuroreceptors to interfere with the nephrotoxicity of cisplatin [47].

*Eclipta prostrata* L. (EP) and *Daucus carota* (DC) belong to the same family of composites. The plants are characterized by the presence of an array of phytochemicals including alkaloids, glycosides, coumarins, flavonoids and sterols [1,48]. EP and DC have been traditionally used for blackening, promoting hair growth, and strengthening the hair. In Ayurveda medicine, the leaf extract is considered a powerful liver tonic and rejuvenator. Pharmacological activities of EP and DC include analgesic activity, anti-aggression activity, anti-bacterial activity, anticancer activity, anti-diabetic activity, anti-helminthic activity, hepatoprotective activity, anti-inflammatory activity, hair growth promoter activity. Besides these, EP and DC have nootropic potential, they enhance memory and learning [49–52]. Pretreatment with hydroalcoholic extract of EP and DC significantly increases the levels of superoxide dismutase, glutathione peroxidase, reduced glutathione, catalase, glutathione-S-transferase, glutathione reductase, and decreases MDA in the brain. EP and DC at higher doses markedly reduced ischemic neuronal loss of the rat brain induced by occluding bilateral common carotid arteries for 30 min, followed by 4 h of reperfusion [53].

However, in the present study, the synergistic effects of cisplatin with DC and EP plant extracts were checked at 400 and 600 mg/kg administration. They showed a significant effect against cis-induced nephrotoxicity, but the mechanism of action is not fully understood. Many hypotheses suggests that they are involved in the reduction of inflammation, oxidative stress, or apoptosis. The present study results revealed that Cis group significantly reduced the bodyweight of rats by increasing the kidney weight. The animal weight loss in Cis group was strongly related to insufficient nutrition, an increase in metabolic processes, metabolic imbalances, or mental conflict in the CDDP treatment community [54]. In addition, cisplatin-induced tubular necrosis raised kidney weight in groups treated with cisplatin due to ischemia or proliferation [55]. The animals treated with Cis + DC/Cis + EP (600 mg/kg) showed a substantial reduction in kidney weight due to their anti-inflammatory effects [56]. Similar results were observed by Singh et al. [57], which revealed that co-administration of Cis (30 mg/kg) + morin hydrate (40 mg/kg) significantly reduced the Cis-treated rats’ kidney weight as compared to Cis group. Similarly, Sahu et al. [58] reported that supplementation of Cis + Bai at the rate of 50 mg/kg significantly decreased the relative kidney weight of about 1.3 g and increased the body weight as compared to Cis group in rats. They also observed a significant reduction in serum creatinine levels of about 0.5 mg/dL, which was nearly equal to the control.

The present study showed that co-administration of Cis + DC/Cis + EP extract at the rate of 600 mg/kg significantly (p < 0.001) improved renal function. Results revealed that 600 mg/kg supplementation of Cis + DC/Cis +EP successfully (p < 0.005) reduced the urinary creatinine, sodium (Na), and potassium (K) levels up to the control as compared to the Cis group that increased the urinary creatinine, Na and K levels after a single dose of 5 mg/kg cisplatin. In comparison, 600 mg/kg co-administration of Cis + DC/Cis + EP improved (p < 0.005) the plasmin creatinine, Na, and K levels that were reduced after intake of cisplatin. The weakening of membrane pumps like sodium-potassium is due to the nephrotoxicity caused by cisplatin. It causes a decrease in sodium reabsorption, that’s why their concentration in urine is increased [59,60]. In the present study, the cisplatin-treated group displayed hyponatremia and hypokalemia. Co-administration of Cis + DC/Cis + EP (400 and 600 mg/kg) allows sodium and potassium levels to increase near normal values relative to the Cis groups. Thus, impediments of hyponatremia and hypokalemia can be allocated to the protective action of Cis + DC/Cis + EP (p < 0.005) in Cis-induced nephrotoxicity.

Results revealed that the best outcomes were noted after the 7^th^ days of an experiment compared to the 14^th^ and 21^st^ days of observation. Our results are in agreement with the findings of Chtourou et al. [61]. They revealed that co-administration of Cis + Nar100 significantly reduced the serum creatinine level up to 0.47 ± 0.02 mg/dL in rats as compared to Cis group that showed a serum level of 0.97 ± 0.02 mg/dL after 5 mg/kg administration. Similarly, they observed that co-administration of Cis + naringin (100 mg/kg) increased the urine creatinine level of 6.27 ± 0.92 mg/dL compared to Cis group (4.05 ± 0.12 mg/dL). Fatima et al. [62] demonstrated that co-administration of Cis + A20 (EGCG + CoQ10) reduced the serum creatinine level of 1.36 ± 0.30 mg/dL as compared to Cis group (3.13 ± 0.25 mg/dL), urine Na, K, Ca^2+^, and Mg^2+^ levels of 110 ± 2.56, 28 ± 3.01, 4.83 ± 0.05, and 27.4 ± 2.2 µmol/24 h, respectively. Similarly, a study revealed that administration of wogonin of 50 mg/kg/day significantly reduced the serum creatinine and blood urea nitrogen (BUN) levels up to the control in Cis-treated rats [63]. Vasaikar et al. [64] stated that a 40 mg/kg dose of D-pinitol successfully reduced urine and serum creatinine levels by 0.82 0.03 and 1.16 0.09 mg/dL, respectively, when compared to the Cis group, which increased urine and serum creatinine levels to 2.06 0.25 and 6.21 0.12 mg/dL, respectively.

## Conclusions

Overall results of the present study revealed that the co-administration of Cis + DC extract and Cis + EP extract at the rates of 400 and 600 mg/kg significantly improved renal function by reducing urinary creatinine, urine output, urine flow rate, Na and K and elevating the plasmin creatinine, Na and K levels in cisplatin-induced nephrotoxicity in albino Wister rats. It is observed that Cis + EP ata rate of 600 mg/kg showed better results as compared to Cis + DC extract. The physical, histopathological, and biochemical analysis of both extracts supports the nephroprotective improvement in rats. Moreover, Cis + DC/Cis + EP might be utilized as a potent drug against cisplatin-induced nephrotoxicity in the near future. However, promising profile of Cis + DC/Cis + EP needs to be thoroughly investigated in upcoming studies for a proper understanding of the mechanism of action of these extracts. Further studies are recommended for intervention and determining the exact nephroprotective potential phytochemicals in DC and EP extracts.

## List of abbreviation

EP, *Eclipta prostrata*; DC, *Daucus carota*; BW, body weight; CVS, cardiovascular system; Cis, cisplatin; i.p, intraperitoneal; ROS, reactive oxygen species; GFR, glomerular filtration rate; PCT, proximal convoluted tubules; PBS, phosphate buffer saline; and AKI, acute kidney injuries.
